# Characteristics of and risk factors for epilepsy after autoimmune and infectious encephalitis

**DOI:** 10.1186/s12883-026-04680-4

**Published:** 2026-02-07

**Authors:** Judith N. Wagner, Tobias Moser, Joachim Gruber, Eirini Mylonaki, Vincent Böhm, Daniel Schwarzenhofer, Anna R. Tröscher, Eva Lenzenweger, Ingomar Krehan, Eva Söllradl, Markus Leitinger, Raimund Helbok, Eugen Trinka, Tim J. von Oertzen

**Affiliations:** 1 University Hospital Essen, Essen, Germany; 2 Department of Neurology, Evangelisches Klinikum Gelsenkirchen, Teaching Hospital University Duisburg-Essen, Munckelstrasse 27, Gelsenkirchen, 45879 Germany; 3 Department of Neurology, Neurocritical Care, and Neurorehabilitation, Christian Doppler University Hospital, Paracelsus Medical University and Centre for Cognitive Neuroscience, European Reference Network EpiCARE, Salzburg, Austria; 4 Department of Neurology, Kepler University Hospital, Johannes Kepler University Linz, Linz, Austria; 5 Clinical Research Institute of Neuroscience, Kepler University Hospital, Johannes Kepler University Linz, Linz, Austria; 6 Neuroscience Institute, Centre for Cognitive Neuroscience, Christian Doppler University Hospital, Paracelsus Medical University, Salzburg, Austria; 7 Medical Directorate, University Hospital Würzburg, Würzburg, Germany

**Keywords:** Encephalitis, Inflammation, Infection, Epilepsy, Seizures

## Abstract

**Introduction:**

Encephalitis is associated with significant morbidity including epilepsy. Data on post-encephalitic epilepsy (PEE) incidence and optimal management remain scarce. We prospectively investigated the frequency, characteristics, and risk factors of PEE after autoimmune encephalitis (AE) and infectious encephalitis (IE) in three Austrian tertiary care hospitals over ten years.

**Materials & methods:**

We included patients ≥ 18 years diagnosed according to internationally recognized encephalitis algorithms or verification of well-characterized antineuronal autoantibodies (AE) or pathogens (IE). Epilepsy diagnosis followed International League Against Epilepsy criteria. All available clinical data were collected from electronic patient files. A prospective follow-up was conducted via structured interviews and clinical visits. Statistical analyses included Kaplan-Meier plots, correlation analyses, and regression models.

**Results:**

Of 149 patients, 26 (17%) had AE, 108 (73%) IE, and 15 (10%) encephalitis of unknown etiology. Prospective follow-up was achieved in 74% of cases. Median follow-up was 2304 days (IQR 1433; 3274). Acute symptomatic seizures (ASS), acute symptomatic status epilepticus and PEE are significantly more frequent in AE than in IE (*p* < 0.001). Antiseizure medication (ASM) use persisted in 85% of AE and 30% of IE patients at last follow-up, despite only 12% meeting PEE criteria. In both types of encephalitis, we see a shift from focal-to-bilateral semiology. Difficult-to-treat seizures during initial hospitalization correlated with PEE risk (rho = 0.317; *p* < 0.001).

**Discussion:**

Our study provides insights into PEE characteristics, management, and prognosis. It highlights the prevalence of ASM overuse in the wake of encephalitis and provides a prognostic tool assessing the individual PEE risk.

**Supplementary Information:**

The online version contains supplementary material available at 10.1186/s12883-026-04680-4.

## Introduction

Encephalitis is an inflammation of the brain caused by infectious or autoimmunological etiologies. The disease leads to tremendous morbidity including persisting focal neurological deficits, as well as cognitive, and neuropsychiatric symptoms [1, 2]. Furthermore, encephalitis is frequently associated with acute symptomatic seizures (ASS), status epilepticus, and post-encephalitic epilepsy (PEE) [3–7]. Cerebral inflammation can in fact not only elicit epileptic seizures, but also trigger and perpetuate epileptogenesis [8]. Among the various mechanisms mediating epileptic seizures and epileptogenesis are neuronal loss and gliosis, microglial and astrocyte activation leading to cytokine release and upregulation of the complement system, blood-brain barrier leakage, remodeling of the extracellular matrix, and altered transmitter metabolism [9, 10].

Few data exist concerning on the incidence of PEE and its risk factors. Moreover, the optimal therapeutic approach remains a matter of debate. Out of 1425 results obtained from a 2018 PubMed review using the search term “encephalitis AND epilepsy,” only three studies specifically focus on epilepsy resulting from encephalitis across various causes in adulthood over a 12-year period starting from the first description of the NMDA-receptor as an antigen of autoimmune encephalitis (AE) [11–14]. Only one of these was prospective [13], and only one included patients in central Europe [12]. Most studies in this field focus on a pediatric population or on a single encephalitis etiology and primarily comprise cohorts from Asia or North America. Thus, no prospective data exist for the encephalitis spectrum encountered in Central Europe for the investigated period and it remains unclear, whether the existing results can be generalized to various populations and different pathogens.

Retrospective studies are susceptible to overdiagnosis of PEE because ASS occurring during the acute phase or a recurrence of AE activity are often taken as indicative of PEE, although they do not meet the International League Against Epilepsy (ILAE) criteria of epilepsy [15, 16]. On the other hand, underdiagnosis of PEE may occur if the diagnosis is made from retrospective analyses of patient files but without sufficient clinical information. Furthermore, in those studies conducted before the widespread implementation of auto-antibody diagnostics, a high percentage of cases with unknown or “nonspecific” causes was included which probably corresponded to AE [13]. Hence, an update on this topic is warranted.

In order to analyze diagnosis, treatment, and prophylaxis of PEE, we identified all patients diagnosed with encephalitis during a period of 10 years in three tertiary care hospitals in Austria and carried out a prospective follow-up. We aimed to describe demographic characteristics of the population, as well as the frequency, characteristics, and risk factors of epilepsy after AE and infectious encephalitis (IE). We also develop a prognostic tool to estimate the individual PEE risk.

## Materials & methods

### Study population and data extraction

All patients with an ICD 10 diagnosis consistent with encephalitis (Supplementary File 1) who were treated at the Departments of Neurology 1 and 2 of Kepler University Hospital Linz (Austria) and Christian Doppler University Hospital, Paracelsus Medical University, Salzburg (Austria) between January 1st 2007 and July 31th 2017 were screened. The two departments in Linz are located at different sites and serve different catchment areas of the city of Linz and the State of Upper Austria. The study was approved by the ethics committee of Upper Austria (EK Nr. 1112/2018) and Salzburg (EK Nr. 415-E/2419/4-2018).

Patients with the following inclusion criteria were analyzed:

- Age 18 to 99 years
- At least “possible” encephalitis according to the International Encephalitis Consortium diagnostic criteria [17], verification of well-characterized antineuronal autoantibodies or pathogens or at least “probable” autoimmune encephalitis according to Graus et al. [18]

Exclusion criteria:

- Severe pre-encephalitis comorbidity (modified Ranking Scale (mRS) > 3) with relevant influence on the clinical presentation.
- Preexisting epilepsy.
- Lack of follow-up.

Patient demographics, disease specific factors, hospital complications and outcome were extracted from the electronic patient file (Supplementary File 1). Groups were categorized as AE or IE according to the discharge diagnosis. If a definite diagnosis could not be made at the time of discharge, the patient file including initial hospital stay and all follow-ups were reviewed by an experienced neurologist. Further diagnostic results (particularly magnetic resonance imaging (MRI), lumbar puncture, microbiological diagnostics, auto-antibody assays) as well as the clinical course (monophasic vs. remittent) were considered to assign patients to AE or IE. Patients in whom the etiology of encephalitis could not be specified as AE or IE were diagnosed as encephalitis of unknown etiology (EUE). Seizures were defined as ASS if there was evidence of active encephalitic disease. The diagnosis was made on clinical grounds, as no clear timeframe has been defined for seizures occurring in connection with encephalitis [19]. Status epilepticus was defined as generalized convulsions during five minutes or more (ten minutes for other seizure semiologies). Non-convulsive status was diagnosed according to the Salzburg Consensus Criteria [20].

### Prospective follow-up

All patients were informed about the trial and the planned follow-up by letter. If the patient consented, a structured telephone interview was conducted identifying persisting neurological deficits, the mRS score [21], events suggestive of epileptic seizures, concomitant disease, antiseizure medication (ASM) in the interval, among others (Supplementary File 2). All patients were screened for epileptic seizures using a validated questionnaire [22, 23]. Diagnosis of epilepsy was made according to the ILAE criteria [16]:

1. At least two unprovoked (or reflex) seizures occurring >24 h apart
2. One unprovoked (or reflex) seizure and a probability of further seizures similar to the general recurrence risk (at least 60%) after two unprovoked seizures, occurring over the next 10 years (based upon EEG and/or MRI findings)
3. Diagnosis of an epilepsy syndrome

PEE was defined as continuing epileptic seizures despite of sufficient anti-infectious or immunosuppressive treatment. If the patient was unavailable for the interview, information was sought from a close relative or the patient’s last general practitioner. The patient’s last documented phone number was called at least three times at different times on different days before the patient was declared lost to follow-up.

During the telephone interview, patients were invited for a clinical visit in one of the participating hospitals. They were instructed to bring a caregiver if possible. This visit was conducted by an experienced neurologist and included a detailed patient and third-party history, a neurological examination and evaluation of the current functional status (mRS; Supplementary File 3). Further examinations such as electroencephalography (EEG), laboratory tests or cognitive screening tests were performed as needed in case of diagnostic uncertainty.

### Statistics

All data of continuous variables were evaluated for normal distribution (test of normality: Kolmogorov-Smirnov with Lilliefors significance correction, type I error = 10%). Continuous variables with normally distributed data were compared by the t-test for independent samples. For comparisons of continuous variables without normally distributed data and of variables measured on ordinal scales, the exact Mann-Whitney U test was used. Dichotomous variables were compared by the Fisher’s exact test, the other categorical variables by the exact chi-square test.

A Kaplan Meier plot depicted the occurrence of PEE. Two-sided 95% confidence intervals (CI) were calculated for one- to ten-year incidences of PEE according to Clopper-Pearson.

Correlations between dichotomous variables and variables measured on ordinal scales as well as (always not normally distributed) continuous variables were reviewed by point biserial Spearman’s rank correlation coefficients. Associations of dichotomous variables were investigated by Phi coefficients (combined with Fisher’s exact test).

The predictability of PEE was investigated by logistic regression analysis with the independent variables age, etiology of encephalitis, ASS, status epilepticus, cortical lesion on MRI, number of ASM, mechanical ventilation, epileptic discharges on EEG (all variables refer to the initial hospitalization due to encephalitis).

The type I error was not adjusted for multiple testing. Therefore, the results of inferential statistics are descriptive only. Statistical analyses were performed using the open-source R statistical software package, version 4.2.3 (The R Foundation for Statistical Computing, Vienna, Austria).

## Results

### Study population

Of the 160 patients screened, 149 patients (46% female) fulfilled the inclusion criteria and were included in the final analysis. AE was diagnosed in 25 (17%) and IE in 109 (73%). Fifteen individuals (10%) were classified as EUE. Patient characteristics, etiologies, and treatment strategies are shown in Table 1. Characteristics of EUE patients were more similar to IE than to AE patients. 74% had a prospective follow up: a telephone interview in 101 and a clinical visit in 33 patients of whom seven were accompanied by a caregiver. Median duration of follow-up from first symptoms was 1761 days for AE (IQR 1058–2244) and 2585 days for IE (IQR 1459–3335). More data from this population were collected for a different project [24]. 

**Table 1:** Patient characteristics in the AE and IE cohort

	Autoimmune encephalitis (*N* = 26)		Infectious encephalitis (*N* = 108)		
**Age at onset (years; median, range)**	59 (21–77)		63 (20–92)		*p* = 0.072
**Delay to hospitalization (days; median**,** IQR)**	2 (0–76)		2 (0–5)		*p* = 0.273
**Length of hospitalization (days; median**,** IQR)**	22 days (IQR 11–52)		20 days (IQR 14–39)		*p* = 0.953
**Admitted to ICU** (*N*; %)	7; 27		45; 42		*p* = 0.186
**Mechanical ventilation** (*N*; %)	5; 20		22; 21		*p* > 0.999
**Modified Rankin Score ≤ 1 at discharge** (*N*; %)	10;40 (n.d. in 1 patient)		45; 43 (n.d. in 3 patients)		*p* = 0.983
**Presenting symptoms**					
** Disorders of consciousness** (*N*; %)	15; 58		108; 100		*p* < 0.001
** Cognitive deficits** (*N*; %)	21; 84 (n.d. in 1 patient)		96; 89		*P* = 0.501
** Focal neurological deficits** (*N*; %)	6; 23		60; 56		*p* = 0.004
** Acute symptomatic seizures** (*N*; %)	22; 85		22; 20		*p* < 0.001
** Fever > 38 °C** (*N*; %)	2; 8		77; 72		*p* < 0.001
** Headache** (*N*; %)	4; 15		66; 61		*p* < 0.001
** CSF leukocytes (n/µl; median**,** range)**	6 (0; 11)		125 (52; 347)		*p* < 0.001
Immunosuppressive treatment* (*N*; %)	20; 81		15;14		
		N**		**N**	
	High-dose steroids	15	High-dose steroids	15	
*during initial hospitalization	Intravenous immunoglobulins	14	Intravenous immunoglobulins	3	
**11 patients received > 1 substance	Plasmapheresis	5			
	Rituximab	1			
**Etiology** (*n*)	CASPR2 (5)		TBEV (26)		
	LGI1 (4)		HSV-1 (9)		
	NMDAR (2)		VZV (2)		
	SOX1/Amphiphysin (1)		EBV (2)		
	GABAB (2)		Influenza A virus (2)		
	GAD (1)		Measles virus (1)		
	Ma2 (1)		CMV (1)		
	AMPA (1)		Enterovirus (1)		
	Seronegative (9)		Streptococcus pneumoniae (10)		
			Listeria monocytogenes (4)		
			Neisseria meningitidis (2)		
			Streptococcus – other (4)		
			Staphylococcus aureus (2)		
			Other / not determined (42)		

Abbreviations: *n.d.* not documented, *IQR*  interquartile range

### Imaging characteristics

Fluid-attenuated inversion recovery (FLAIR)-/ T2-weighted cerebral MRI showed lesions in 13 (50%) of patients with AE and 30 (28%) patients with IE (*p* = 0.037) in the acute phase. All MRI-positive AE patients exhibited cortical lesions, while these were present in only 16 (15%) of all IE cases. Three (13%) and 12 patients (12%), respectively, had contrast-enhancing lesions. The most common lesion sites were mesio-temporal (92%), supratentorial extratemporal (23%), basal ganglia (15%), and latero-temporal (15%) in AE and mesio-temporal (55%), supratentorial extratemporal (41%), basal ganglia (31%), latero-temporal (28%), and infratentorial (24%) in IE. The difference for the frequency of mesio-temporal lesions was significant (*p* < 0.031). Presence of MRI lesions did not correlate with the incidence of PEE (see supplementary file 4). Neither did cortical or mesiotemporal location of these lesions.

### Characteristics of ASS and PEE

**Fig. 1:**
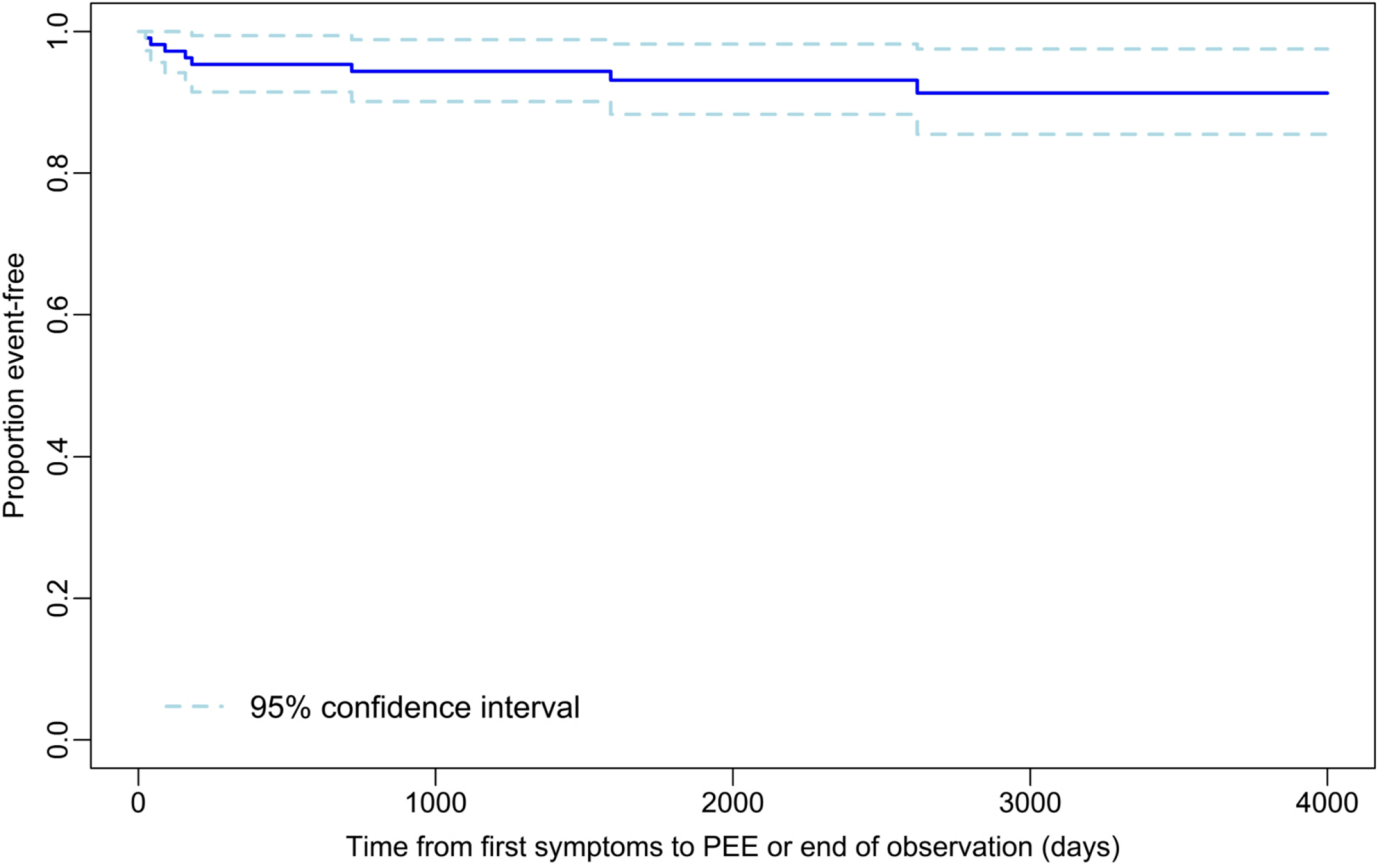
Kaplan-Meier-Plot of time to first PEE-defining seizure in IE patients

ASS occurred more frequently in AE (*p* < 0.001; Table 2) and did not correlate with age at onset of encephalitis (correlation coefficient = -0.069; *p* = 0.400). The semiology of ASS was most frequently focal to bilateral (motor, non-motor) in both causes of encephalitis. Eight patients had two different types of ASS. Status epilepticus was commonly diagnosed in individuals with AE (*n* = 7; 27%), while it only occurred in three (3%) IE patients (*p* < 0.001). An EEG study was available for 134 patients (90%) during their initial hospital stay. In 13 (50%) of AE and 14 (14%) of IE patients, EEG showed epileptic discharges and/ or ictal patterns (*p* < 0.001). PEE was diagnosed in 18 patients (12%), with significant preponderance of AE patients (*p* < 0.001; Tables 2 and 3). The median interval between symptom onset and PEE-diagnosis in AE was 39 days (range 0-602; well within the 25th percentile (1058 days) of the follow-up period). In IE, the first seizure defining PEE occurred at a median of 169 days (range 24-2620) after symptom onset (for Kaplan-Meier plot see Fig. 1). Due to the challenge in determining the exact onset of PEE in AE, no Kaplan-Meier analysis was performed in this cohort. Seizure type in PEE was most frequently focal aware in AE and focal to bilateral (motor, non-motor) in IE. Status epilepticus was rare in the interval between hospital discharge and last follow-up.

**Table 2:** Semiology of acute symptomatic seizures and seizures in post-encephalitic epilepsy

	AE	IE	
Acute symptomatic seizures (N; %)*	22; 85	22; 20	*p* < 0.001
Focal to bilateral (N; %)	11; 50	16; 73	*p* = 0.215
Focal aware (N; %)	9; 41	2; 9	*p* = 0.034
Focal with impaired awareness (N; %)	7; 32	7; 32	*p* > 0.999
Status epilepticus (N; %)	7; 27	2; 3	*p* < 0.001
**Post-encephalitic epilepsy (**N; %)*	10; 39	8; 7	*p* < 0.001
Focal to bilateral (N; %)	4; 40	6; 75	*p* = 0.17
Focal aware (N; %)	8; 80	4; 50	*p* = 0.377
Focal with impaired awareness (N; %)	3; 30	1; 13	*p* = 0.338
Status epilepticus (N; %)*	1; 4	1; 1	*p* = 0.361

*percentages relative to entire AE or IE cohort; remaining percentages relative to subcohort (AE – acute symptomatic seizures, AE – PEE; IE – acute symptomatic seizures, IE – PEE)

**Table 3:** Characteristics of PEE patients

Age-range (yrs)	Sex	Etiology	Type last FU	ASS (1 = yes, 2 = no)	SE (1 = yes, 2 = no)	FND (1 = yes, 2 = no)	CSF-PL (1 = yes, 2 = no)	MRI -L (1 = yes, 2 = no)	ETP (1 = yes, 2 = no)	No. ASM (*n*)	MV (1 = yes, 2 = no)	mRS
70–79	m	IE (Suspected bacterial)	T	2	2	1	1	2	2	0	2	4
70–79	f	IE (HSV1)	eF	2	2	2	2	1	1	1	1	5
70–79	m	IE (TBEV)	eF	2	2	1	1	1	2	1	2	1
70–79	m	AE (anti-LGI1)	V	1	2	2	2	1	1	1	2	1
70–79	f	AE (anti-SOX1, anti-amphiphysin)	T	1	1	1	2	1	2	4	2	3
60–69	m	AE (anti-GABAB)	eF	1	2	2	1	2	2	0	2	1
60–69	m	AE (anti-CASPR2)	V	1	2	2	2	1	1	1	2	1
30–39	f	AE (seronegative)	V	1	1	1	1	1	1	7	1	3
20–29	m	AE (seronegative)	V	1	1	1	1	1	1	5	1	3
70–79	f	AE (anti-CASPR2)	T	1	2	2	2	1	2	1	2	2
30–39	f	IE (Streptococcus pneumoniae)	eF	1	2	1	1	1	1	2	1	4
60–69	f	AE (GABAB)	eF	1	2	2	1	2	1	1	2	2
70–79	f	IE (HSV1)	T	2	2	1	2	1	1	1	2	3
40–49	m	IE (suspected Influenza A)	T	2	2	1	1	2	2	0	2	0
40–49	f	IE (Streptokokkus pyogenes)	T	2	2	1	1	1	n.d.	n.d.	1	5
30–39	f	AE (seronegative)	T	1	1	1	1	1	1	2	1	5
30–39	f	AE (anti-NMDAR)	T	1	2	1	2	1	2	0	2	0
70–79	f	IE (Streptococcus pneumoniae)	T	2		1	1	1	n.d.	1	1	

Abbreviations: *n.d.* no data, *AE* autoimmune encephalitis, *ASM* anti-seizure medication, *ASS* acute symptomatic seizure, *CSF-PL* CSF pleocytosis, *eF* electronic patient file, *ETP* epileptiform discharge, *FND* focal neurological deficit, *FU* follow-up, *HSV* herpes simplex virus, *IE* infectious encephalitis, *MRI-L* MRI lesion, *mRS* modified Rankin Scale, *MV* mechanical ventilation, *SE* status epilepticus, *TBEV* tick-borne encephalitis virus, *T* telephone interview, *V* clinical visit. Acute symptomatic seizures, status epilepticus and number of ACDs refer to initial presentation, mRS to initial discharge

During the initial hospital stay, significantly more AE patients (20; 77%) than IE patients (26; 26%) received at least one ASM for the indication of ‘epileptic seizures’ (*p* < 0.001). The median number of ASM per patient during the initial hospital stay was 1 (range 0–7) in AE and 0 (range 0–5) in IE. At last follow-up, 22 patients with AE (85%) and 32 with IE (30%) were on ASM. The median number of ASM per patient at that time was one drug per patient (range 0–4) in AE and 0 drugs (range 0–2) in IE (*p* < 0.001). For the frequency, dosage, and efficacy of substances used at both time points see Table 4. For immunosuppressive therapy see Table 1. The median delay from symptom onset to the initiation of immunosuppressive therapy in AE was 35.5 days (IQR 6–92), with a median delay of two days from hospitalization to treatment commencement (IQR 0–76).

**Table 4:** Characteristics of ASM therapy in PEE patients

		AE				IE			
		LEV	LCM	PHE	VAL	LEV	LCM	PHE	VAL
**Initial hospitalization**	Patients (*n*)	18	6	3	3	25	1	4	3
	Max. dose* (mg/d)	2500 (1000–4000)	400 (300–600)	500 (350–750)	2400 (750–3000)	2000 (1000–4000)	400 (n.a.)	700 (375–2400)	2760 (1500–6000)
	Improvement**	67%	67%	33%	33%	68%	100%	75%	100%
**Last follow-up**	Patients (*n*)	14	8	0	3	10	1	0	3
	Max. dose* (mg/d)	2750 (1000–5000)	400 (150–600)		3000 (1000–3600)	1500 (750–3000)	100 (n.a.)		500 (200–1500)
	Improvement**	86%	75%		50%	100%	100%		33%

Abbreviations: *LEV* levetiracetam, *LCM * lacosamide, *PHE* phenytoin, *VAL* valproate, *n.a.* not applicable

*median (range)

**> 50% seizure reduction

The probability of developing PEE correlated positively with the number of ASMs during the initial hospital stay (rho = 0.317; *p* < 0.001). The diagnosis of PEE positively correlated with the mRS score at last contact (*p* = 0.007), but neither did the presence of acute symptomatic seizures nor the necessity of mechanical ventilation. Logistic regression analysis did not reveal a significant association between the diagnosis of PEE and all other variables tested in AE and IE patients (for a complete list of these variables refer to supplementary file 4). Multivariate logistic regression revealed a significant effect of encephalitis etiology on PEE risk only when controlling for age, presence of ASS or status epilepticus, cortical lesions on MRI, number of ASM, mechanical ventilation or presence of epileptiform discharges (*p* = 0.014). None of the other factors showed a significant effect.

We developed a prognostic tool to estimate an individual’s risk of developing PEE based on results of routine clinical and paraclinical tests. This tool is provided as an interactive Excel file (Supplementary File 5). The cut-off can be adjusted to obtain the desired sensitivity and specificity. ROC-analysis indicated an optimal cut-off of 0.139 to distinguish between those patients likely to develop a PEE from those that are not (sensitivity 81%, specificity 81%; Youden Index 0.6206).

## Discussion

### ASS and PEE in encephalitis – definition and epidemiology

ASS are a common symptom, particularly in AE [25, 26], reflecting frequent involvement of highly epileptogenic mesio-temporal structures. PEE was a relatively rare sequela. This confirms earlier findings reporting drug-resistant epilepsy in only 16% of patients one year after the onset of AE [27]. However, it is important to consider the possibility of underreporting of seizures [28]. Furthermore, patients who actually had epilepsy but were well-controlled on ASM on follow-up may falsely have been classified as epilepsy-free. PEE may also have been underdiagnosed as only 22% of patients received in-person follow-ups. However, inquiring about the presence of unprovoked seizures is the most important part in the diagnosis of PEE according to ILAE criteria. To improve history-taking in this context, we recurred to a questionnaire shown to have high specificity and sensitivity for seizure detection. In instances of diagnostic doubt, e.g. in a patient with ictal events that could not be classified as epileptic or non-epileptic by history-taking alone, EEG was performed at the discretion of the investigator.

A major challenge during follow-up of encephalitis patients who experienced seizures during the subacute or chronic phase of the disease consists in differentiating AE-associated epilepsy from AE recurrence. Additionally, clinicians must distinguish between patients with PEE who remain seizure-free on ASM therapy and those without epilepsy, i.e., those patients in whom the PEE diagnosis needs to be reconsidered. Rada and Bien addressed this issue by proposing a definition for AE-associated epilepsy, which distinguishes between AE with high risk of seizure recurrence without ASM or epilepsy surgery (such as AE with antibodies against intracellular antigens) and AE associated with antibodies against extracellular antigens, characterized by a high likelihood of seizure freedom after immunotherapy [15, 29, 30]. In the latter group, epilepsy can be diagnosed if seizures persist for at least two years after immunotherapy initiation, if there are no signs of encephalitis on MRI, no hypermetabolism in fluorodeoxyglucose positron emission tomography, the cerebrospinal fluid (CSF) cell count is normal, and antibody titers have substantially decreased. These criteria were published in 2023 and could not be used prospectively for our study. Hence, for some patients there is a lack of information if they do or do not meet these criteria (e.g. for a lack of titre determination during the course of treatment). However, they align well with our clinical approach to the diagnosis of PEE in this specific context.

### ASS and PEE in encephalitis – risk factors and characteristics

The risk factors for PEE after AE in a recent meta-analysis include coma, status epilepticus, cranial MRI abnormalities, and focal EEG abnormalities at disease onset, as well as longer time to immunotherapy [31]. Another study additionally describes increased risk of seizure recurrence for females in NMDAR encephalitis [32]. The latter study also reports a significantly lower PEE prevalence in their cohort consisting exclusively of patients with AE caused by neuronal surface antibodies compared to our mixed AE cohort. This consolidates the view of AE associated with antibodies directed against intracellular antigens as a further PEE risk factor. However, these risk factors have only inconsistently been replicated in other studies, including our own analysis. This inconsistency may be attributed to the retrospective design of all but one study, as well as to the use of an imprecise PEE definition, which may limit the specificity of this diagnosis. Other explanatory factors include lack of standardization of MRI and EEG acquisition and the heterogeneity of patient cohorts, as different PEE risk factors have been observed in AE of varying etiology [33, 34].

Difficult to treat seizures at disease onset of AE have been identified as a prognostic factor for PEE [35]. This tendency is reflected in our cohort, where the number of ASM administered during initial hospitalization correlated positively with PEE incidence on univariate – albeit not on multivariate – analysis. Although epileptic discharges and ictal patterns were frequently observed on EEG, particularly in AE, their presence did not predict PEE. This lack of association has been demonstrated previously for the occurrence of early seizures and for PEE [11, 13, 34, 36, 37].

While young age has previously been associated with a higher incidence of seizures in AE [36], we did not observe this trend in our cohort, potentially due to the mitigating effect to the higher age of IE patients or because we only included adults.

While cortical and mesio-temporal MRI lesions were more frequent in AE compared to IE, they were not predictive of PEE. While this contradicts earlier findings, it supports results from studies on ASS in encephalitis [25, 37, 38]. Lesions occurring during the acute phase of the encephalitis may be reversible – particularly in AE with antibodies directed against neural surface structures – and may be the consequence rather than the cause of epileptic seizures [15, 39]. Hence, in this type of encephalitis, MRI changes may not be associated with the PEE risk as has been described before [32–34].

In both AE and IE, there is a considerable risk of ASS progressing from focal to bilateral [25, 26, 36, 40]. In PEE, focal seizures become more predominant. This shift could indicate a partial reversal of initial inflammatory processes and the formation of glial scars [10]. Alternatively, a selection bias may exist: nearly 50% of patients with LGI1-antibody-positive encephalitis have been reported to experience exclusively focal seizures, predominantly faciobrachial dystonic seizures [36]. A significant proportion of this AE subtype among PEE patients would therefore contribute to an increased prevalence of focal seizures.

We observed multiple cases of status epilepticus as a presenting symptom of AE. This finding aligns with studies on the etiology of new-onset status epilepticus (NOS; [41]), which highlight AE as an important differential diagnosis in patients presenting with status epilepticus. AE subtypes appear to vary in terms of the associated risk of status epilepticus, with a higher incidence in NMDAR encephalitis than in GAD- or LGI1-antibody positive patients [36]. The requirement for ICU treatment and mechanical ventilation was prevalent in both AE and IE. Some studies have linked these features to a poorer outcome [42, 43] and high prevalence of ASS [40]. However, we did not observe a correlation with the incidence of PEE or the overall outcome as measured by the mRS score.

### ASS and PEE in encephalitis – therapy and outcome

Polytherapy with ASM was common in patients hospitalized for AE. This points towards a high proportion of ASM-resistant seizures and is consistent with findings that rigorous immunosuppression is the most important therapeutic approach in AE [44]. Levetiracetam was the most frequently prescribed ASM in both groups, with doses falling within recommended ranges. Older ASM such as phenytoin or valproic acid were rarely used, mainly in patients with status epilepticus, and tended to have been discontinued or tapered by the last follow-up. This stands in stark contrast to studies conducted in other geographic regions, where valproic acid remains the most common ASM [26]. No high-level data exist as to which ASM is most effective in PEE. Limited evidence suggests a potential superiority of sodium channel inhibitors in PEE after AE [45, 46]. However, we observed high rates of seizure remission among patients treated with levetiracetam during both initial hospitalization and last follow-up.

Current ASMs did not show to prevent epilepsy after an acute brain insult. Hence the recommendation is to give it only over a short period [47, 48]. Current practices often deviate from this [49]^,^ [50]. This was also the case in our series. Thus, if prescribed, ASM use should be regularly reevaluated after abatement of the acute stage of the disease. This reassessment does not seem to be consistently conducted in clinical practice, as evidenced by the fact that 54 of our patients were still on ASM at last follow-up, despite only 18 patients meeting the criteria for PEE. On the other hand, all PEE patients from our cohort were still on ASM at the last available follow-up. Therefore, despite the significant interval between the acute disease and the first PEE-defining seizure, overdiagnosis seems to present a substantially larger challenge than underdiagnosis. The optimal timing to start tapering ASM after encephalitis cannot be deduced from our data. Previous trials have reported no difference in seizure outcome between an early and late withdrawal group (on ASM ≤ or > 3 months) in AE [51]. This casts doubt on a definition of PEE based on the duration of ASM treatment [11, 52].

### Limitations

Limitations of this study include the challenge in precisely pinpointing the occurrence of the first PEE-defining seizure, particularly in AE, and to distinguish ASS from unprovoked seizures that warrant the diagnosis of PEE. As there are no clear guidelines as to the timeframe within which to assume ongoing encephalitic activity, this decision must be made purely on clinical grounds. This definition contains some measure of subjectivity. Average follow-up was long, helping in acquiring more certainty as to the correct classification of seizures since the likelihood of ASS diminishes with time. In total, we pursued a very conservative approach to the diagnosis of seizures defining epilepsy. Therefore, PEE prevalence is more likely to be under- than overestimated in our study.

Another cause of a potential underestimate of PEE prevalence resides in the challenge to distinguish PEE patients with a stable seizure control on ASM from those that do not suffer from epilepsy in the first place. To obtain an even more precise estimate of the true prevalence, ASM tapering and regular re-evaluation of the patient over several months, preferably one to two years would be required.

Another limitation pertains to the heterogeneity of our patient cohort due to diverse etiologies in both AE and IE. This may be one factor explaining the lack of association of PEE with suspected risk factors. Due to regionality, TBE-cases are overrepresented in our cohort. Nonetheless, these can serve as models for other Flavivirus infections – such as West Nile encephalitis – that rank among the most common viral CNS infections worldwide and with whom they share the tropism for the basal ganglia and the low propensity to cause epilepsy [53]. However, it remains important to consider the local pathogen spectrum when applying our data.

Limitations apply to the comparison of different disease entities – in this case autoimmune and infectious etiology. However, presenting these disorders – that share many clinical and pathophysiological commonalities – side-by-side allows us to extract new epileptological insights.

Another confounder may have been the changing approach towards AE during the years after initial description of NMDAR encephalitis in 2007. Cases of AE might have been misdiagnosed as IE (particularly viral) due to lack of awareness for AE or insufficient diagnostic methods. This would have falsely increased the number of PEE cases in the IE cohort given the higher PEE prevalence in AE. Furthermore, if the PEE cohort consisted of a mixture of insufficiently treated “early” and well-treated “late” AE cases, the true PEE prevalence in a contemporary cohort may be overestimated. However, an analysis of the percentage of AE (relative to total) cases and the number of PEE cases diagnosed per year (for details see supplementary file 6) does not permit to draw these conclusions.

AE associated with CASPR2 and LGI1 antibodies dominated in our sample. This contrasts with other studies where NMDAR encephalitis is the most prevalent AE. As all three have a good prognosis concerning PEE, we postulate that the likelihood of a falsely high PEE rate due to overrepresentation of prognostically worse AE is rather low.

26% of our patients only had retrospective follow-up data. We chose to include these as to avoid bias towards patients with less severe clinical courses as among the main reasons participants could not be contacted prospectively were premature death or severe disability. Potential reduction of diagnostic sensitivity and specificity in this subgroup was offset by an extended follow-up period with a median of more than six years for the entire cohort in conjunction with high epileptological expertise and meticulous documentation in all participating centers.

Lastly, the prognostic tool developed for PEE has only been validated in the derivation cohort. Further validation in an independent cohort is needed.

## Conclusion

This investigation stands out as one of the few studies characterizing PEE applying thorough epileptological expertise and using a prospective approach for most participants. We also developed a prognostic tool for the estimation of the individual PEE risk after AE and IE. This may prove particularly useful for future trials on PEE prevention. Prospective enquiry about possible epilepsy symptoms reduces the risk of underdiagnosis, while allowing reassessment of ambiguous symptoms that may otherwise lead to overdiagnosis of epilepsy.

## Supplementary Information

Supplementary Material 1: Supplementary File 1. ICD-10 codes used to screen for encephalitis patients. Data collected during screening, for initial hospitalization, at retrospective follow-up, and at prospective follow-up via telephone interview and clinical visit.

Supplementary Material 2: Supplementary File 2. Protocol telephone interview.

Supplementary Material 3: Supplementary File 3. Protocol clinical visit.

Supplementary Material 4: Supplementary File 4. Variables showing no association with PEE on logistic regression analysis.

Supplementary Material 5: Supplementary File 5. Prognostication tool for the diagnosis of PEE. Please only enter or modify values in yellow cells, i.e. the cut-off value and the values of the explanatory variables. Do not modify the other cells or the deposited formulas and cross-references. Use the comma for decimal separation.

Supplementary Material 6: Supplementary File 6. Total number of encephalitis cases diagnosed per year 2007 – 2017. Percentage of autoimmune encephalitis cases relative to total cases and total number of PEE cases.

## Data Availability

Data are available upon reasonable request from the corresponding author.
