# Supplementary material for: Characteristics of and risk factors for epilepsy after autoimmune and infectious encephalitis
Source: BMC Neurol. 2026 Feb 7;26:133. doi: 10.1186/s12883-026-04680-4 (PMC12930834; doi:10.1186/s12883-026-04680-4)
Supplement: Supplementary file 2 — Supplementary Material 2: Supplementary File 2. Protocol telephone interview. [file 12883_2026_4680_MOESM2_ESM.docx]

**Clinical visit**

**Screening number Patient:** **__________ Patient ID_______________**

**Date of birth:** **_________ m/f**

**Name of interviewer:______________________ Department/Clinic: ________________**

**Date of interview:** **____________**

**Is the patient accompanied by another person (third-person anamnesis)?** **Yes/No**

**If yes, name and relationship to the patient:** **______________________**

**Step 1**: Has the patient been able to clarify all his/her questions regarding the study and has he/she signed the consent form? **Yes/No**

(if no: clarify what information the patient needs that would allow him/her to participate in the study; if he/she definitely refuses - clarify and document whether the retrospectively collected data may be used)

**Step 2**: Go through the following questionnaires with the patient and their carer (Tables 1, 2 and 3). In the event of divergent answers regarding the telephone interview, **the results of the personal examination are decisive for the final assessment regarding the presence and characteristics of postencephalitic epilepsy!**

**Screening for epileptic seizures (Placencia et al., Brain 1992)**

|  | **Answer (please mark clearly)** | **Personal assessment: the reported event corresponds with ... certainty to an epileptic seizure (please mark clearly)** | **If the event corresponds with HIGH certainty to an epileptic seizure, adde the seizure classification according to the ILAE Seizure Classification 2017 + frequency and period of events + response to therapy + provocation factors** |
| --- | --- | --- | --- |
| Have you ever lost consciousness? | Yes / No | high/low/unclear |  |
| Have you ever had attacks in which you lose contact with the surroundings? | Yes / No | high/low/unclear |  |
| Have you ever had attacks of shaking of the arms or legs which you could not control? | Yes / No | high/low/unclear |  |
| Have you ever had attacks in which you fall to the ground with loss of consciousness? | Yes / No | high/low/unclear |  |
| Have you ever had attacks in which you fall and bite your tongue? | Yes / No | high/low/unclear |  |
| Have you ever had attacks in which you fall and lose control of your bladder? | Yes / No | high/low/unclear |  |
| Have you ever had attacks in which you fall and become pale? | Yes / No | high/low/unclear |  |
| Have you ever had attacks in which you lose your memory for a short period of time? | Yes / No | high/low/unclear |  |
| Have you ever had attacks of strange behaviour and loss of memory? | Yes / No | high/low/unclear |  |
| Have you ever had brief attacks of shaking or trembling in one arm or leg or in the face? | Yes / No | high/low/unclear |  |
| Have you ever had attacks of tingling or numbness which move up your arm, leg or body? | Yes / No | high/low/unclear |  |
| Have you ever had attacks of jerkings which move up your arm, leg or body? | Yes / No | high/low/unclear |  |
| Have you ever had attacks in which you lose contact with the surrounding and experience a feeling of unreality or dreaminess? | Yes / No | high/low/unclear |  |
| Have you ever had attacks in which you lose contact with the surrounding and experience a sensation in which objects change shape or size? | Yes / No | high/low/unclear |  |
| Have you ever had attacks in which you lose contact with the surrounding and experience abnormal visions? | Yes / No | high/low/unclear |  |
| Have you ever had attacks in which you lose contact with the surrounding and experience abnormal sounds? | Yes / No | high/low/unclear |  |
| Have you ever had attacks in which you lose contact with the surroundings and experience abnormal smells? | Yes / No | high/low/unclear |  |
| Have you ever had attacks in which you behave momentarily in a confused fashion? | Yes / No | high/low/unclear |  |
| Have you ever had attacks of palpitation? | Yes / No | high/low/unclear |  |
| Have you ever been told that you have or had epilepsy or epileptic seizures? | Yes / No | high/low/unclear |  |

**Further questions – Table 2**

|  | **Antwort (bitte eindeutig markieren)** | **Detailinformationen** |
| --- | --- | --- |
| If you answered "yes" to one of the questions in Table 1 (Screening according to Placencia et al., Brain 1992) - has a diagnosis already been made in this regard? | Yes / No | Which diagnosis? |
| If you answered "yes" to one of the questions in Table 1 (Screening according to Placencia et al., Brain 1992), have further diagnostic tests (e.g. EEG, cMRI, LP) already been carried out? | Yes / No | Which tests? Results? |
| In the case of existing epileptic seizures, collect further detailed information for each type of seizure |  | Semiology:  First-time occurrence:  Frequency (n/week): |
| Are you currently taking medication for epileptic seizures (collect detailed information for each drug)? | Yes / No | Name of drug:  Max. dose:  Start of medication:  Efficacy:  Side effects:  Reasons for potential dose reductions: |
| Have you taken any other medication for epileptic seizures in the interval since the last follow-up at our clinic (collection of detailed information for each drug)? | Yes / No | Name of drug:  Max. dose:  Start of medication:  Efficacy:  Side effects:  Reasons for discontinuation: |
| Have you ever had status epilepticus?  (If necessary, explain to patient: particularly long seizure, not self-limiting...) | Yes / No | Semiology:  When:  Duration:  Therapy (where):  Therapy (how): |
| According to the patient, is there a residual neurological deficit (outside of epilepsy)? | Yes / No | Which deficit: |
| According to the patient, has a new cerebral structural lesion occurred in the interval since the last follow-up? | Yes / No | Which lesion: |
| Does the patient have a new concomitant disease that has occurred in the interval since the last follow-up? | Yes / No | Which disease: |
| Is the patient taking immunosuppressive therapy for the indication encephalitis? | Yes / No | Name of drug:  Dose:  Start of medication: |

**Modified Rankin Scale**

0  The patient has no residual symptoms.

1  The patient has no significant disability; able to carry out all pre-stroke activities.

2  The patient has slight disability; unable to carry out all pre-stroke activities but able to look after self without daily help.

3  The patient has moderate disability; requiring some external help but able to walk without the assistance of another individual.

4  The patient has moderately severe disability; unable to walk or attend to bodily functions without assistance of another individual.

5  The patient has severe disability; bedridden, incontinent, requires continuous care.

6  The patient has expired

**Physical examination**

|  | **Answer (please mark clearly)** | **If the answer is "yes" - please specify** |
| --- | --- | --- |
| Is the patient disorientated in terms of person, place or time? | Yes / No |  |
| Does the patient suffer from a disorder of consciousness? | Yes / No |  |
| Is there an abnormal mood? | Yes / No |  |
| Is there a speech disorder? | Yes / No |  |
| Are there indications of a disorder of other higher brain functions? | Yes / No |  |
| Is there an oculomotor disorder? | Yes / No |  |
| Is there a disorder of the other cranial nerves? | Yes / No |  |
| Is there a paresis? | Yes / No |  |
| Is there a sensory disorder? | Yes / No |  |
| Is there an abnormality in the reflex status/are there pathological reflexes? | Yes / No |  |
| Is there a coordination disorder/ataxia/dysmetria? | Yes / No |  |
| Is there a history of vegetative dysfunction? | Yes / No |  |
| Is there a stance and gait disorder (specify aids if necessary)? | Yes / No |  |
| **Final assessment of the neurological syndrome syndrome** | |  |

**Final assessment of the interviewer:**

- The patient most likely suffers from postencephalitic epilepsy - yes/no (please mark clearly)
- The first seizure defining postencephalitic epilepsy occurred on ________ (date)
- Please also note the information from the retrospective data collection ant the telephone interview!
- Further investigations were carried out or scheduled for definitive categorisation (yes/no; if yes: list investigations (e.g. EEG, cMRI, …)
