# Supplementary material for: Characteristics of and risk factors for epilepsy after autoimmune and infectious encephalitis
Source: BMC Neurol. 2026 Feb 7;26:133. doi: 10.1186/s12883-026-04680-4 (PMC12930834; doi:10.1186/s12883-026-04680-4)
Supplement: Supplementary file 3 — Supplementary Material 3: Supplementary File 3. Protocol clinical visit. [file 12883_2026_4680_MOESM3_ESM.docx]

**Logistic regression analysis did not reveal a significant association between the diagnosis of PEE and these variables:**

- Length of interval between first symptoms and hospitalization
- Sex and age at initial manifestation of encephalitis
- In the case of AE: length of interval between first symptoms and initiation of immunosuppressive therapy
- Length of first encephalitis-related hospital stay
- Presence of disorders of consciousness at initial presentation
- Presence of disorders of focal neurological deficits at initial presentation
- MRI/CT-lesions caused by encephalitis
- Cortical lesions on MRI/CT caused by encephalitis
- Mesiotemporal lesions on MRI/CT caused by encephalitis
- Presence of acute symptomatic seizures or status epilepticus at initial presentation
- Epileptic discharges on EEG performed during the initial hospital stay
- Presence of an immunocompromising disease
- Mechanical ventilation during initial hospitalization
- CSF pleocytosis
- Study center
