# Supplementary material for: Characteristics of and risk factors for epilepsy after autoimmune and infectious encephalitis
Source: BMC Neurol. 2026 Feb 7;26:133. doi: 10.1186/s12883-026-04680-4 (PMC12930834; doi:10.1186/s12883-026-04680-4)
Supplement: Supplementary file 5 — Supplementary Material 5: Supplementary File 5. Prognostication tool for the diagnosis of PEE. Please only enter or modify values in yellow cells, i.e. the cut-off value and the values of the explanatory variables. Do not modify the other cells or the deposited formulas and cross-references. Use the comma for decimal separation. [file 12883_2026_4680_MOESM5_ESM.docx]

**RELEVANT ICD 10 CODES**

G04.0, G04.8, G04.9, G05.1, G05.2, G05.8, A81.1/2/8/9, A83.0-9, A84.0/1/8/9, A85.0, A85.1, A85.2, A85.8, A86, A89, A97.2/9, B00.4, B05.0, B26.2, B06.0, B01.1, B02.0, B25.88, J10.8, J09, B05.1, A02.0, A17.0, A17.8, A32.1, A87.-, G00.-, G01.-, G02.-, G03.-, G37.8

**DATA COLLECTD_SCREENING**

- Age
- Sex
- Alteration of consciousness
- Fever > 38°C
- Acute symptomatic seizures
- New neurological deficits
- CSF leukocytosis
- New MRI or CT lesions compatible with encephalitis
- EEG alterations compatible with encephalitis
- Exclusion of relevant differential diagnoses

**DATA COLLECTD_INITIAL HOSPITALIZATION**

- Date of birth
- Sex
- Date first symptoms
- Date first hospitalization
- Date extraction patient data
- Etiology encephalitis
- Definite diagnosis (specific pathogen or antibody)
- Certainty of diagnosis
- Interval first symptoms – initiation of therapy (for AE)
- Level of consciousness
- New neurological deficit (yes vs. no; if yes – which)
- Other symptoms (headache, autonomous symptoms, neuropsychological symptoms)
- MRI/CT lesion compatible with encephalitis (contrast-enhancement? Gray vs. white matter? Topographic localization? Cerebral edema?)
- Structural lesion independent of encephalitis (which? Potentially epileptogenic?)
- Acute symptomatic seizures (semiology?)
- Status epilepticus (semiology?)
- EEG: epileptiform discharges? Seizure patterns?
- Antiseizure medication (which? Maximal dose? Effect on seizure activity?)
- Antibacterial/ antiviral/ immunosuppressive therapy
- Comorbidity, particularly immunosuppressive disease
- Need for ICU-treatment or mechanical ventilation
- Date first CSF analysis
- Results first CSF analysis (pleocytosis, leukocyte count, leukocyte differentiation, oligoclonal bands, intrathecal immunoglobulin synthesis, CSF/serum glucose ratio, protein)
- Highest serum CRP
- Lowest thrombocyte count
- Lowest serum sodium
- TSH
- mRS score at discharge

**DATA COLLECTD_RETROSPECTIVE FOLLOW-UP**

- Date of birth
- Date last follow-up
- Date of death if patient deceased
- Date extraction patient data
- Neurological deficit (yes vs. no; if yes – which)
- Status epilepticus (semiology?)
- Antiseizure medication (which? Maximal dose? Effect on seizure activity? Side effects? Duration of therapy/ ongoing?)
- Immunosuppressive therapy (which? Maximal dose? Duration of therapy/ ongoing?)
- Postencephalitic epilepsy (PEE; first PEE-defining seizure, semiology, frequency)
- New structural lesion independent of encephalitis
- Encephalitis relapse or ongoing disease
- mRS score

**DATA COLLECTD_PROSPECTIVE FOLLOW-UP: TELEPHONE INTERVIEW**

- Date follow-up
- Date of death if patient deceased
- Neurological or cognitive deficit (yes vs. no; if yes – which)
- Postencephalitic epilepsy (PEE; first PEE-defining seizure, semiology, frequency)
- Status epilepticus (semiology?)
- Antiseizure medication (which? Dose? Effect on seizure activity? Side effects? Duration of therapy/ ongoing?)
- Immunosuppressive therapy
- New structural lesion independent of encephalitis
- New relevant morbidity
- mRS score

**DATA COLLECTD_PROSPECTIVE FOLLOW-UP: CLINICAL VISIT**

- Date follow-up
- Neurological or cognitive deficit (yes vs. no; if yes – which; results of physical neurological examination)
- Postencephalitic epilepsy (PEE; first PEE-defining seizure, semiology, frequency)
- Status epilepticus (semiology?)
- Antiseizure medication (which? Dose? Effect on seizure activity? Side effects? Duration of therapy/ ongoing?)
- Immunosuppressive therapy
- New structural lesion independent of encephalitis
- New relevant morbidity
- mRS score
- presence of caregiver
