# Supplementary material for: Characteristics of and risk factors for epilepsy after autoimmune and infectious encephalitis
Source: BMC Neurol. 2026 Feb 7;26:133. doi: 10.1186/s12883-026-04680-4 (PMC12930834; doi:10.1186/s12883-026-04680-4)
Supplement: Supplementary file 6 [file 12883_2026_4680_MOESM6_ESM.docx]

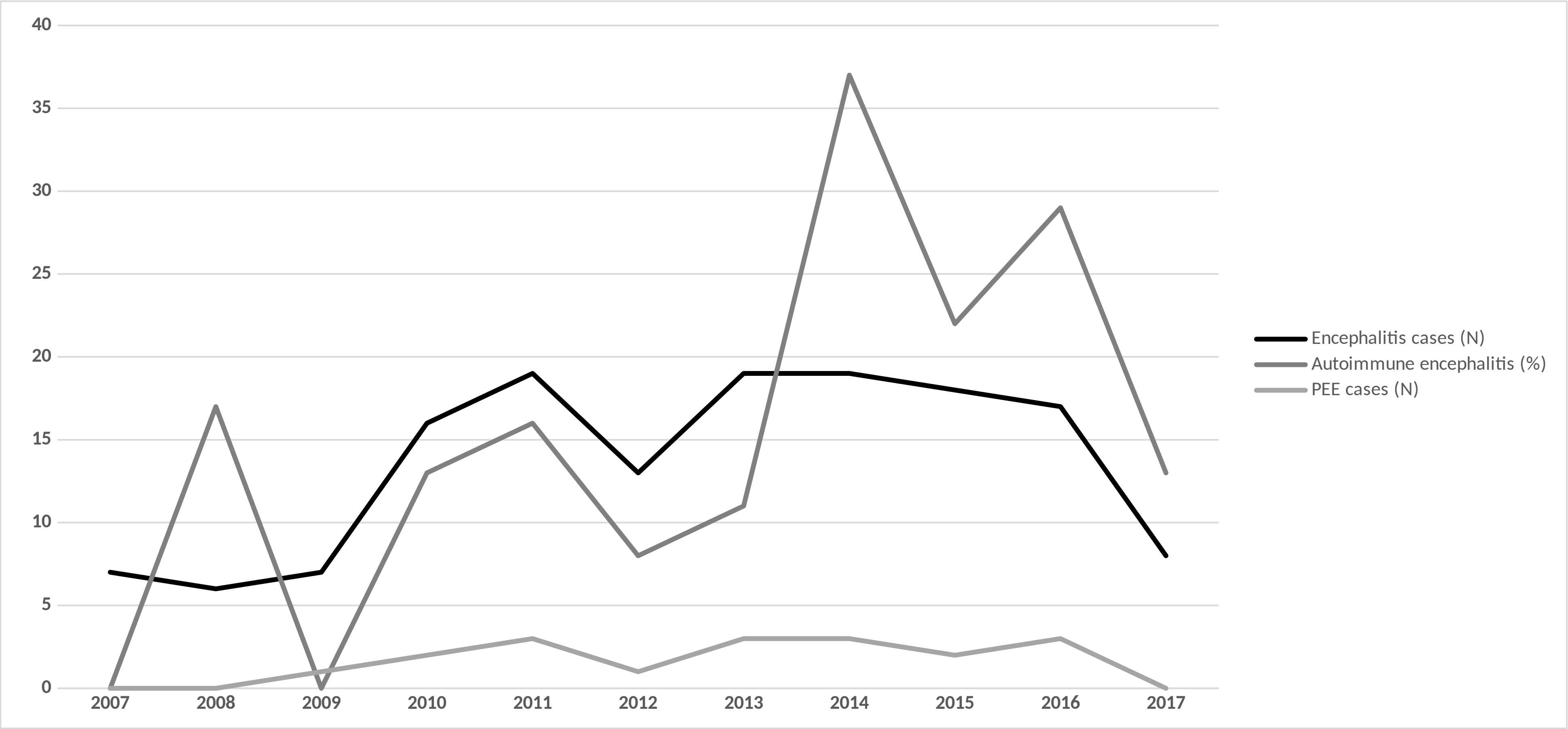

40
35
30
25
20
15
10
5
0
2007
2008
2009
2010
2011
2012
2013
2014
2015
2016
2017
Encephalitis cases (N)
Autoimmune encephalitis (%)
PEE cases (N)
